# Supplementary material for: Comparative Proteomics Study on the Postharvest Senescence of Volvariella volvacea
Source: J Fungi (Basel). 2022 Aug 4;8(8):819. doi: 10.3390/jof8080819 (PMC9410126; doi:10.3390/jof8080819)
Supplement: Supplementary file 1 [file jof-08-00819-s001.zip › jof-1770245-supplementary.pdf]

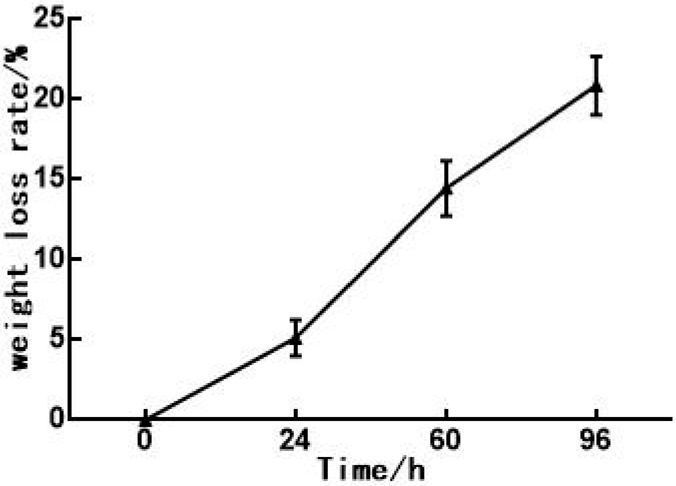

Figure S1: Weight loss rate of *V. volvacea* fruiting bodies stored at 15 °C.

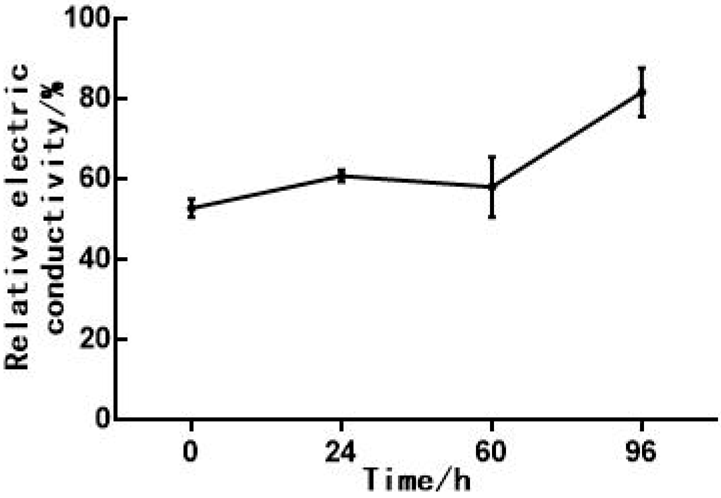

Figure S2: Relative electric conductivity of *V. volvacea* fruiting bodies stored at 15 °C.

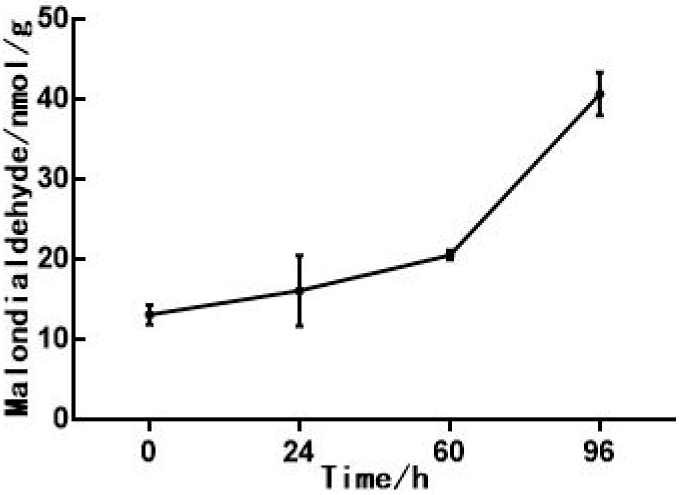

Figure S3: Malondialdehyde content of *V. volvacea* fruiting bodies stored at 15 °C.
